# (4-Carb­oxy-2-sulfonato­benzoato-κ^2^ *O* ^1^,*O* ^2^)bis­(1,10-phenanthroline-κ^2^ *N*,*N*′)manganese(II)

**DOI:** 10.1107/S1600536810023743

**Published:** 2010-06-26

**Authors:** Qiong-Fang Wu, Ming-Xing Li

**Affiliations:** a Department of Chemistry, College of Science, Shanghai University, Shanghai 200444, People’s Republic of China

## Abstract

In the title complex, [Mn(C_8_H_4_O_7_S)(C_12_H_8_N_2_)_2_], the Mn^II^ atom is chelated by one 4-carb­oxy-2-sulfonato­benzoate anion and two phenathroline (phen) ligands in a distorted octa­hedral MnN_4_O_2_ geometry. The benzene ring of the 4-carb­oxy-2-sulfonato­benzoate anion is twisted with respect to the two phen ring systems at dihedral angles of 66.38 (9) and 53.56 (9)°. In the crystal, inter­molecular O—H⋯O and C—H⋯O hydrogen bonding links the mol­ecules into chains running parallel to [100]. Inter­molecular π–π stacking is also observed between parallel phen ring systems, the face-to-face distance being 3.432 (6) Å.

## Related literature

The 4-carb­oxy-2-sulfonato­benzoate anion has been used to construct coordination polymers through both carboxyl and sulfonate groups, see: Horike *et al.* (2006 ▶); Xiao *et al.* (2007 ▶).
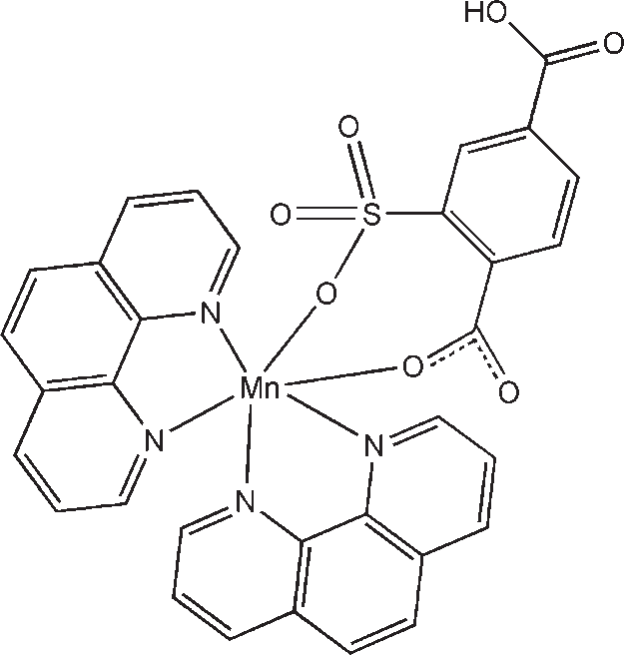


## Experimental

### 

#### Crystal data

[Mn(C_8_H_4_O_7_S)(C_12_H_8_N_2_)_2_]
*M* *_r_* = 659.52
Triclinic, 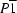

*a* = 9.490 (2) Å
*b* = 9.688 (2) Å
*c* = 16.842 (4) Å
α = 73.294 (4)°
β = 89.016 (4)°
γ = 70.159 (3)°
*V* = 1389.6 (5) Å^3^
*Z* = 2
Mo *K*α radiation
μ = 0.61 mm^−1^
*T* = 298 K
0.35 × 0.22 × 0.18 mm

#### Data collection

Bruker SMART APEXII CCD diffractometer
Absorption correction: multi-scan (*SADABS*; Bruker, 2001 ▶) *T*_min_ = 0.815, *T*_max_ = 0.898
6937 measured reflections
4883 independent reflections
4344 reflections with *I* > 2σ(*I*)
*R*_int_ = 0.017

#### Refinement

*R*[*F* ^2^ > 2σ(*F* ^2^)] = 0.039
*wR*(*F* ^2^) = 0.109
*S* = 1.07
4883 reflections
407 parameters
1 restraint
H-atom parameters constrained
Δρ_max_ = 0.47 e Å^−3^
Δρ_min_ = −0.57 e Å^−3^

### 

Data collection: *APEX2* (Bruker, 2007 ▶); cell refinement: *SAINT* (Bruker, 2007 ▶); data reduction: *SAINT*; program(s) used to solve structure: *SHELXS97* (Sheldrick, 2008 ▶); program(s) used to refine structure: *SHELXL97* (Sheldrick, 2008 ▶); molecular graphics: *ORTEP-3 for Windows* (Farrugia, 1997 ▶); software used to prepare material for publication: *SHELXL97*.

## Supplementary Material

Crystal structure: contains datablocks I, global. DOI: 10.1107/S1600536810023743/xu2781sup1.cif

Structure factors: contains datablocks I. DOI: 10.1107/S1600536810023743/xu2781Isup2.hkl

Additional supplementary materials:  crystallographic information; 3D view; checkCIF report

## Figures and Tables

**Table 1:** Selected bond lengths (Å)

Mn1—O1	2.0769 (15)
Mn1—O3	2.1878 (15)
Mn1—N1	2.2824 (17)
Mn1—N2	2.3321 (18)
Mn1—N3	2.2592 (17)
Mn1—N4	2.2458 (18)

**Table 2:** Hydrogen-bond geometry (Å, °)

*D*—H⋯*A*	*D*—H	H⋯*A*	*D*⋯*A*	*D*—H⋯*A*
O7—H7⋯O2^i^	0.82	1.72	2.517 (3)	165
C8—H8⋯O6^ii^	0.93	2.42	3.209 (4)	142
C18—H18⋯O5^iii^	0.93	2.46	3.306 (3)	151

Symmetry codes: (i) 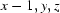; (ii) 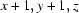; (iii) 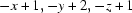.

**Fig. 1.**
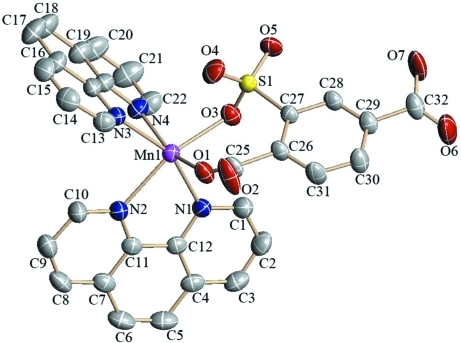
Molecular structure of the title compound.

## supplementary crystallographic information

### Comment

Aromatic acids have extensively been used to prepare coordination complexes because of the high affinity of carboxylate function and the versatile coordination modes of the carboxylate groups. Among the aromatic acids, 2-sulfoterephtalate (stp) ligand is useful in the construction of new metal–organic coordination polymers due to its two carboxylate groups and one sulfonate group (Horike *et al.*, 2006; Xiao *et al.*, 2007). Herein, we present a new six-coordinated manganese(II) complex based on stp. The molecular structure of the title compound is shown in Fig.1. The coordination geometry of the Mn(II) is distorted octahedral, in which four positions are occupied by four N atoms of two chelating phen ligands and the other two occupied by two O atoms from one caboxylate and one sulfonate of one stp ligand. The Mn—N distances are in the range 2.2458 (18) - 2.3321 (18) Å. The Mn—O distances are 2.0769 (15) and 2.1878 (15)°. The adjacent molecules are linked by O—H···O intermolecular hydrogen bonds to form a one dimensional chain structure. Furthermore, π-π stacks between phen ligands from adjacent molecules link these chains forming a two dimensional layer.

### Experimental

A mixture of MnCl_2_.4H_2_O (39.6 mg, 0.2 mmol), NaH_2_sta (53.6 mg, 0.2 mmol), phen (90.2 mg, 0.5 mmol) and 8.0 ml of distilled water was placed in a Teflon-lined stainless steel vessel and heated at 403 K for 3 day. After slow cooling to room temperature, yellow block crystals were obtained in 35% yield (based on NaH_2_stp).

### Refinement

H atoms were positioned geometrically and refined as riding atoms with C—H = 0.93 and O—H = 0.82 Å, U_iso_(H) = 1.2U_eq_(C) and 1.5U_eq_(O).

### Crystal data

**Table:**

[Mn(C_8_H_4_O_7_S)(C_12_H_8_N_2_)_2_]	*Z* = 2
*M**_r_* = 659.52	*F*(000) = 674
Triclinic, *P*1	*D*_x_ = 1.576 Mg m^−^^3^
Hall symbol: -P 1	Mo *K*α radiation, λ = 0.71073 Å
*a* = 9.490 (2) Å	Cell parameters from 1000 reflections
*b* = 9.688 (2) Å	θ = 2.3–28.1°
*c* = 16.842 (4) Å	µ = 0.61 mm^−^^1^
α = 73.294 (4)°	*T* = 298 K
β = 89.016 (4)°	Block, yellow
γ = 70.159 (3)°	0.35 × 0.22 × 0.18 mm
*V* = 1389.6 (5) Å^3^	

### Data collection

**Table:**

Bruker SMART APEXII CCD diffractometer	4883 independent reflections
Radiation source: fine-focus sealed tube	4344 reflections with *I* > 2σ(*I*)
graphite	*R*_int_ = 0.017
φ and ω scan	θ_max_ = 25.2°, θ_min_ = 2.3°
Absorption correction: multi-scan (*SADABS*; Bruker, 2001)	*h* = −11→8
*T*_min_ = 0.815, *T*_max_ = 0.898	*k* = −11→11
6937 measured reflections	*l* = −20→16

### Refinement

**Table:**

Refinement on *F*^2^	Primary atom site location: structure-invariant direct methods
Least-squares matrix: full	Secondary atom site location: difference Fourier map
*R*[*F*^2^ > 2σ(*F*^2^)] = 0.039	Hydrogen site location: inferred from neighbouring sites
*wR*(*F*^2^) = 0.109	H-atom parameters constrained
*S* = 1.07	*w* = 1/[σ^2^(*F*_o_^2^) + (0.0726*P*)^2^ + 0.1828*P*] where *P* = (*F*_o_^2^ + 2*F*_c_^2^)/3
4883 reflections	(Δ/σ)_max_ = 0.001
407 parameters	Δρ_max_ = 0.47 e Å^−^^3^
1 restraint	Δρ_min_ = −0.56 e Å^−^^3^

### Special details

**Table:**

Geometry. All e.s.d.'s (except the e.s.d. in the dihedral angle between two l.s. planes) are estimated using the full covariance matrix. The cell e.s.d.'s are taken into account individually in the estimation of e.s.d.'s in distances, angles and torsion angles; correlations between e.s.d.'s in cell parameters are only used when they are defined by crystal symmetry. An approximate (isotropic) treatment of cell e.s.d.'s is used for estimating e.s.d.'s involving l.s. planes.
Refinement. Refinement of *F*^2^ against ALL reflections. The weighted *R*-factor *wR* and goodness of fit *S* are based on *F*^2^, conventional *R*-factors *R* are based on *F*, with *F* set to zero for negative *F*^2^. The threshold expression of *F*^2^ > σ(*F*^2^) is used only for calculating *R*-factors(gt) *etc*. and is not relevant to the choice of reflections for refinement. *R*-factors based on *F*^2^ are statistically about twice as large as those based on *F*, and *R*- factors based on ALL data will be even larger.

### Fractional atomic coordinates and isotropic or equivalent isotropic displacement parameters (Å^2^)

**Table:**

	*x*	*y*	*z*	*U*_iso_*/*U*_eq_	
Mn1	0.81303 (3)	0.62093 (3)	0.736333 (18)	0.03308 (12)	
S1	0.56855 (5)	0.51204 (6)	0.67785 (3)	0.03438 (15)	
O1	0.89461 (15)	0.38212 (16)	0.77751 (10)	0.0426 (4)	
O2	0.99042 (17)	0.1388 (2)	0.78338 (16)	0.0720 (6)	
O3	0.58796 (16)	0.60968 (16)	0.72599 (10)	0.0419 (3)	
O4	0.68197 (19)	0.4882 (2)	0.62139 (10)	0.0553 (4)	
O5	0.41600 (17)	0.56635 (19)	0.64207 (10)	0.0492 (4)	
O6	0.3320 (2)	0.0085 (2)	0.89832 (14)	0.0753 (6)	
O7	0.23792 (19)	0.1821 (2)	0.77621 (14)	0.0731 (6)	
H7	0.1642	0.1563	0.7867	0.110*	
N1	0.75417 (18)	0.6487 (2)	0.86416 (10)	0.0360 (4)	
N2	1.00468 (19)	0.6749 (2)	0.79177 (11)	0.0377 (4)	
N3	0.93871 (19)	0.6367 (2)	0.62082 (10)	0.0359 (4)	
N4	0.7026 (2)	0.8611 (2)	0.65126 (11)	0.0409 (4)	
C1	0.6354 (3)	0.6297 (3)	0.90112 (14)	0.0454 (5)	
H1	0.5646	0.6122	0.8715	0.055*	
C2	0.6108 (3)	0.6344 (3)	0.98204 (15)	0.0501 (6)	
H2	0.5251	0.6211	1.0055	0.060*	
C3	0.7132 (3)	0.6585 (3)	1.02633 (14)	0.0481 (6)	
H3	0.6996	0.6590	1.0811	0.058*	
C4	0.8391 (2)	0.6827 (2)	0.98951 (13)	0.0401 (5)	
C5	0.9479 (3)	0.7165 (3)	1.03036 (15)	0.0518 (6)	
H5	0.9377	0.7201	1.0848	0.062*	
C6	1.0646 (3)	0.7433 (3)	0.99153 (16)	0.0519 (6)	
H6	1.1319	0.7690	1.0188	0.062*	
C7	1.0876 (2)	0.7330 (2)	0.90899 (14)	0.0407 (5)	
C8	1.2103 (3)	0.7554 (3)	0.86739 (16)	0.0484 (6)	
H8	1.2783	0.7844	0.8918	0.058*	
C9	1.2297 (3)	0.7346 (3)	0.79113 (16)	0.0511 (6)	
H9	1.3114	0.7480	0.7629	0.061*	
C10	1.1254 (3)	0.6930 (3)	0.75616 (15)	0.0458 (5)	
H10	1.1411	0.6767	0.7044	0.055*	
C11	0.9859 (2)	0.6951 (2)	0.86801 (12)	0.0340 (4)	
C12	0.8563 (2)	0.6745 (2)	0.90764 (12)	0.0339 (4)	
C13	1.0580 (2)	0.5287 (3)	0.60794 (14)	0.0432 (5)	
H13	1.0930	0.4332	0.6482	0.052*	
C14	1.1337 (3)	0.5519 (3)	0.53646 (15)	0.0513 (6)	
H14	1.2197	0.4744	0.5305	0.062*	
C15	1.0812 (3)	0.6879 (3)	0.47598 (15)	0.0517 (6)	
H15	1.1297	0.7038	0.4275	0.062*	
C16	0.9538 (3)	0.8046 (3)	0.48635 (14)	0.0463 (5)	
C17	0.8901 (3)	0.9513 (3)	0.42570 (16)	0.0628 (7)	
H17	0.9320	0.9708	0.3752	0.075*	
C18	0.7714 (3)	1.0617 (3)	0.43993 (16)	0.0652 (8)	
H18	0.7325	1.1564	0.3993	0.078*	
C19	0.7035 (3)	1.0363 (3)	0.51642 (15)	0.0508 (6)	
C20	0.5795 (3)	1.1480 (3)	0.53439 (17)	0.0662 (8)	
H20	0.5381	1.2451	0.4960	0.079*	
C21	0.5203 (3)	1.1138 (3)	0.60796 (17)	0.0673 (8)	
H21	0.4373	1.1867	0.6204	0.081*	
C22	0.5843 (3)	0.9696 (3)	0.66442 (16)	0.0545 (6)	
H22	0.5416	0.9478	0.7145	0.065*	
C23	0.7610 (2)	0.8935 (2)	0.57700 (12)	0.0380 (5)	
C24	0.8874 (2)	0.7739 (2)	0.56166 (12)	0.0363 (4)	
C25	0.8833 (2)	0.2560 (2)	0.78317 (14)	0.0385 (5)	
C26	0.7340 (2)	0.2323 (2)	0.79683 (13)	0.0345 (4)	
C27	0.5951 (2)	0.3317 (2)	0.75298 (12)	0.0308 (4)	
C28	0.4700 (2)	0.2891 (2)	0.76675 (13)	0.0348 (4)	
H28	0.3795	0.3531	0.7356	0.042*	
C29	0.4756 (2)	0.1545 (2)	0.82534 (14)	0.0384 (5)	
C30	0.6098 (2)	0.0612 (3)	0.87331 (16)	0.0477 (6)	
H30	0.6137	−0.0260	0.9161	0.057*	
C31	0.7366 (2)	0.0984 (3)	0.85731 (16)	0.0471 (6)	
H31	0.8272	0.0322	0.8878	0.057*	
C32	0.3415 (2)	0.1064 (3)	0.83696 (16)	0.0459 (5)	

### Atomic displacement parameters (Å^2^)

**Table:**

	*U*^11^	*U*^22^	*U*^33^	*U*^12^	*U*^13^	*U*^23^
Mn1	0.03048 (19)	0.03700 (19)	0.03069 (19)	−0.01261 (14)	0.00304 (13)	−0.00756 (14)
S1	0.0258 (3)	0.0387 (3)	0.0326 (3)	−0.0097 (2)	−0.00071 (19)	−0.0034 (2)
O1	0.0307 (8)	0.0364 (8)	0.0575 (9)	−0.0123 (6)	−0.0042 (7)	−0.0083 (7)
O2	0.0222 (8)	0.0439 (9)	0.151 (2)	−0.0099 (7)	0.0107 (10)	−0.0330 (11)
O3	0.0327 (8)	0.0383 (8)	0.0519 (9)	−0.0108 (6)	−0.0001 (6)	−0.0109 (7)
O4	0.0495 (10)	0.0676 (11)	0.0433 (9)	−0.0189 (9)	0.0170 (7)	−0.0111 (8)
O5	0.0350 (8)	0.0501 (9)	0.0493 (9)	−0.0120 (7)	−0.0151 (7)	0.0023 (7)
O6	0.0592 (12)	0.0700 (13)	0.0938 (15)	−0.0415 (10)	0.0020 (11)	0.0027 (12)
O7	0.0289 (9)	0.0722 (12)	0.1011 (15)	−0.0260 (9)	−0.0064 (9)	0.0102 (11)
N1	0.0301 (9)	0.0414 (9)	0.0343 (9)	−0.0116 (7)	0.0020 (7)	−0.0092 (8)
N2	0.0344 (9)	0.0430 (10)	0.0366 (9)	−0.0152 (8)	0.0044 (7)	−0.0113 (8)
N3	0.0336 (9)	0.0411 (9)	0.0333 (8)	−0.0138 (8)	0.0033 (6)	−0.0108 (8)
N4	0.0425 (10)	0.0407 (10)	0.0359 (9)	−0.0115 (8)	0.0067 (8)	−0.0099 (8)
C1	0.0388 (12)	0.0560 (14)	0.0414 (12)	−0.0178 (10)	0.0063 (9)	−0.0132 (11)
C2	0.0450 (13)	0.0572 (14)	0.0450 (13)	−0.0169 (11)	0.0163 (10)	−0.0122 (11)
C3	0.0543 (14)	0.0476 (13)	0.0326 (11)	−0.0073 (11)	0.0060 (10)	−0.0104 (10)
C4	0.0416 (12)	0.0364 (11)	0.0323 (11)	−0.0021 (9)	−0.0028 (9)	−0.0089 (9)
C5	0.0530 (15)	0.0560 (14)	0.0405 (13)	−0.0058 (12)	−0.0063 (11)	−0.0208 (11)
C6	0.0465 (14)	0.0579 (14)	0.0522 (14)	−0.0107 (11)	−0.0104 (11)	−0.0263 (12)
C7	0.0360 (11)	0.0347 (10)	0.0458 (12)	−0.0056 (9)	−0.0069 (9)	−0.0114 (10)
C8	0.0380 (12)	0.0474 (13)	0.0619 (15)	−0.0171 (10)	−0.0083 (11)	−0.0161 (12)
C9	0.0381 (12)	0.0605 (15)	0.0573 (15)	−0.0236 (11)	0.0048 (11)	−0.0140 (12)
C10	0.0406 (12)	0.0589 (14)	0.0428 (12)	−0.0245 (11)	0.0075 (10)	−0.0140 (11)
C11	0.0305 (10)	0.0301 (10)	0.0350 (11)	−0.0043 (8)	−0.0036 (8)	−0.0074 (8)
C12	0.0334 (10)	0.0292 (10)	0.0320 (10)	−0.0045 (8)	−0.0019 (8)	−0.0059 (8)
C13	0.0381 (12)	0.0438 (12)	0.0434 (12)	−0.0092 (10)	0.0002 (9)	−0.0128 (10)
C14	0.0375 (12)	0.0606 (15)	0.0562 (15)	−0.0112 (11)	0.0110 (11)	−0.0256 (13)
C15	0.0488 (14)	0.0632 (15)	0.0464 (14)	−0.0217 (12)	0.0159 (11)	−0.0195 (12)
C16	0.0493 (13)	0.0534 (13)	0.0375 (12)	−0.0212 (11)	0.0118 (10)	−0.0119 (10)
C17	0.0760 (19)	0.0614 (16)	0.0414 (14)	−0.0214 (14)	0.0188 (13)	−0.0049 (12)
C18	0.081 (2)	0.0527 (15)	0.0425 (14)	−0.0143 (14)	0.0095 (13)	0.0031 (12)
C19	0.0591 (15)	0.0430 (13)	0.0409 (13)	−0.0124 (11)	0.0030 (11)	−0.0052 (10)
C20	0.081 (2)	0.0426 (13)	0.0489 (15)	0.0014 (13)	0.0021 (14)	−0.0019 (12)
C21	0.0695 (18)	0.0518 (15)	0.0547 (16)	0.0075 (13)	0.0079 (13)	−0.0119 (13)
C22	0.0542 (15)	0.0508 (14)	0.0454 (13)	−0.0039 (12)	0.0125 (11)	−0.0127 (11)
C23	0.0405 (11)	0.0396 (11)	0.0326 (11)	−0.0148 (9)	0.0026 (9)	−0.0079 (9)
C24	0.0355 (11)	0.0423 (11)	0.0344 (11)	−0.0176 (9)	0.0036 (8)	−0.0112 (9)
C25	0.0213 (9)	0.0388 (11)	0.0514 (13)	−0.0086 (9)	−0.0034 (8)	−0.0093 (10)
C26	0.0236 (10)	0.0334 (10)	0.0440 (11)	−0.0078 (8)	0.0001 (8)	−0.0100 (9)
C27	0.0232 (9)	0.0325 (10)	0.0342 (10)	−0.0076 (8)	0.0018 (7)	−0.0088 (8)
C28	0.0222 (9)	0.0357 (10)	0.0430 (11)	−0.0065 (8)	0.0001 (8)	−0.0107 (9)
C29	0.0266 (10)	0.0348 (10)	0.0526 (13)	−0.0095 (8)	0.0055 (9)	−0.0128 (10)
C30	0.0354 (12)	0.0349 (11)	0.0628 (15)	−0.0121 (9)	0.0012 (10)	0.0000 (11)
C31	0.0288 (11)	0.0376 (11)	0.0615 (15)	−0.0071 (9)	−0.0098 (10)	0.0004 (11)
C32	0.0298 (11)	0.0360 (11)	0.0712 (16)	−0.0117 (9)	0.0082 (11)	−0.0147 (11)

### Geometric parameters (Å, °)

**Table:**

Mn1—O1	2.0769 (15)	C8—C9	1.356 (4)
Mn1—O3	2.1878 (15)	C8—H8	0.9300
Mn1—N1	2.2824 (17)	C9—C10	1.384 (3)
Mn1—N2	2.3321 (18)	C9—H9	0.9300
Mn1—N3	2.2592 (17)	C10—H10	0.9300
Mn1—N4	2.2458 (18)	C11—C12	1.435 (3)
S1—O4	1.4279 (16)	C13—C14	1.393 (3)
S1—O5	1.4375 (15)	C13—H13	0.9300
S1—O3	1.4664 (16)	C14—C15	1.350 (3)
S1—C27	1.777 (2)	C14—H14	0.9300
O1—C25	1.240 (2)	C15—C16	1.396 (3)
O2—C25	1.240 (3)	C15—H15	0.9300
O6—C32	1.211 (3)	C16—C24	1.407 (3)
O7—C32	1.290 (3)	C16—C17	1.425 (4)
O7—H7	0.8200	C17—C18	1.338 (4)
N1—C1	1.321 (3)	C17—H17	0.9300
N1—C12	1.354 (3)	C18—C19	1.425 (4)
N2—C10	1.325 (3)	C18—H18	0.9300
N2—C11	1.353 (3)	C19—C23	1.395 (3)
N3—C13	1.320 (3)	C19—C20	1.400 (4)
N3—C24	1.346 (3)	C20—C21	1.352 (4)
N4—C22	1.319 (3)	C20—H20	0.9300
N4—C23	1.356 (3)	C21—C22	1.380 (4)
C1—C2	1.390 (3)	C21—H21	0.9300
C1—H1	0.9300	C22—H22	0.9300
C2—C3	1.355 (4)	C23—C24	1.437 (3)
C2—H2	0.9300	C25—C26	1.514 (3)
C3—C4	1.396 (3)	C26—C31	1.393 (3)
C3—H3	0.9300	C26—C27	1.404 (3)
C4—C12	1.408 (3)	C27—C28	1.379 (3)
C4—C5	1.425 (3)	C28—C29	1.376 (3)
C5—C6	1.339 (4)	C28—H28	0.9300
C5—H5	0.9300	C29—C30	1.388 (3)
C6—C7	1.430 (3)	C29—C32	1.489 (3)
C6—H6	0.9300	C30—C31	1.370 (3)
C7—C8	1.396 (3)	C30—H30	0.9300
C7—C11	1.397 (3)	C31—H31	0.9300
			
O1—Mn1—O3	87.57 (6)	N2—C11—C7	122.68 (19)
O1—Mn1—N4	159.48 (7)	N2—C11—C12	117.70 (18)
O3—Mn1—N4	83.33 (6)	C7—C11—C12	119.62 (19)
O1—Mn1—N3	94.44 (6)	N1—C12—C4	122.37 (19)
O3—Mn1—N3	117.20 (6)	N1—C12—C11	118.32 (18)
N4—Mn1—N3	73.72 (6)	C4—C12—C11	119.30 (19)
O1—Mn1—N1	94.95 (6)	N3—C13—C14	122.7 (2)
O3—Mn1—N1	84.76 (6)	N3—C13—H13	118.7
N4—Mn1—N1	102.46 (6)	C14—C13—H13	118.7
N3—Mn1—N1	156.45 (6)	C15—C14—C13	119.4 (2)
O1—Mn1—N2	101.05 (6)	C15—C14—H14	120.3
O3—Mn1—N2	155.60 (6)	C13—C14—H14	120.3
N4—Mn1—N2	94.70 (7)	C14—C15—C16	119.8 (2)
N3—Mn1—N2	85.15 (6)	C14—C15—H15	120.1
N1—Mn1—N2	71.87 (6)	C16—C15—H15	120.1
O4—S1—O5	115.77 (11)	C15—C16—C24	117.2 (2)
O4—S1—O3	111.31 (10)	C15—C16—C17	123.7 (2)
O5—S1—O3	110.92 (10)	C24—C16—C17	119.2 (2)
O4—S1—C27	107.73 (10)	C18—C17—C16	121.4 (2)
O5—S1—C27	105.52 (9)	C18—C17—H17	119.3
O3—S1—C27	104.76 (9)	C16—C17—H17	119.3
C25—O1—Mn1	151.70 (14)	C17—C18—C19	121.0 (2)
S1—O3—Mn1	116.54 (8)	C17—C18—H18	119.5
C32—O7—H7	109.5	C19—C18—H18	119.5
C1—N1—C12	117.95 (18)	C23—C19—C20	117.4 (2)
C1—N1—Mn1	125.22 (15)	C23—C19—C18	119.4 (2)
C12—N1—Mn1	116.60 (13)	C20—C19—C18	123.2 (2)
C10—N2—C11	117.11 (18)	C21—C20—C19	119.5 (2)
C10—N2—Mn1	127.55 (15)	C21—C20—H20	120.3
C11—N2—Mn1	115.29 (13)	C19—C20—H20	120.3
C13—N3—C24	118.33 (18)	C20—C21—C22	119.3 (2)
C13—N3—Mn1	126.69 (15)	C20—C21—H21	120.3
C24—N3—Mn1	114.92 (13)	C22—C21—H21	120.3
C22—N4—C23	117.38 (19)	N4—C22—C21	123.7 (2)
C22—N4—Mn1	127.03 (16)	N4—C22—H22	118.2
C23—N4—Mn1	115.51 (13)	C21—C22—H22	118.2
N1—C1—C2	123.3 (2)	N4—C23—C19	122.7 (2)
N1—C1—H1	118.4	N4—C23—C24	117.44 (19)
C2—C1—H1	118.4	C19—C23—C24	119.82 (19)
C3—C2—C1	119.2 (2)	N3—C24—C16	122.51 (19)
C3—C2—H2	120.4	N3—C24—C23	118.39 (18)
C1—C2—H2	120.4	C16—C24—C23	119.1 (2)
C2—C3—C4	119.8 (2)	O1—C25—O2	124.36 (19)
C2—C3—H3	120.1	O1—C25—C26	120.81 (18)
C4—C3—H3	120.1	O2—C25—C26	114.69 (18)
C3—C4—C12	117.4 (2)	C31—C26—C27	117.82 (18)
C3—C4—C5	123.2 (2)	C31—C26—C25	116.49 (18)
C12—C4—C5	119.4 (2)	C27—C26—C25	125.68 (18)
C6—C5—C4	121.0 (2)	C28—C27—C26	119.48 (18)
C6—C5—H5	119.5	C28—C27—S1	116.47 (15)
C4—C5—H5	119.5	C26—C27—S1	124.05 (15)
C5—C6—C7	121.3 (2)	C29—C28—C27	121.75 (18)
C5—C6—H6	119.4	C29—C28—H28	119.1
C7—C6—H6	119.4	C27—C28—H28	119.1
C8—C7—C11	117.7 (2)	C28—C29—C30	119.05 (19)
C8—C7—C6	123.0 (2)	C28—C29—C32	121.21 (19)
C11—C7—C6	119.3 (2)	C30—C29—C32	119.7 (2)
C9—C8—C7	119.7 (2)	C31—C30—C29	119.6 (2)
C9—C8—H8	120.2	C31—C30—H30	120.2
C7—C8—H8	120.2	C29—C30—H30	120.2
C8—C9—C10	118.7 (2)	C30—C31—C26	122.0 (2)
C8—C9—H9	120.7	C30—C31—H31	119.0
C10—C9—H9	120.7	C26—C31—H31	119.0
N2—C10—C9	124.1 (2)	O6—C32—O7	124.7 (2)
N2—C10—H10	118.0	O6—C32—C29	122.5 (2)
C9—C10—H10	118.0	O7—C32—C29	112.8 (2)
			
O3—Mn1—O1—C25	22.0 (3)	C1—N1—C12—C4	−0.6 (3)
N4—Mn1—O1—C25	−41.6 (4)	Mn1—N1—C12—C4	−175.35 (15)
N3—Mn1—O1—C25	−95.1 (3)	C1—N1—C12—C11	179.93 (19)
N1—Mn1—O1—C25	106.6 (3)	Mn1—N1—C12—C11	5.1 (2)
N2—Mn1—O1—C25	179.0 (3)	C3—C4—C12—N1	1.9 (3)
O4—S1—O3—Mn1	−21.97 (13)	C5—C4—C12—N1	−177.40 (19)
O5—S1—O3—Mn1	−152.41 (9)	C3—C4—C12—C11	−178.60 (18)
C27—S1—O3—Mn1	94.20 (10)	C5—C4—C12—C11	2.1 (3)
O1—Mn1—O3—S1	−52.43 (10)	N2—C11—C12—N1	−5.4 (3)
N4—Mn1—O3—S1	109.14 (10)	C7—C11—C12—N1	175.34 (18)
N3—Mn1—O3—S1	41.31 (11)	N2—C11—C12—C4	175.11 (18)
N1—Mn1—O3—S1	−147.63 (10)	C7—C11—C12—C4	−4.2 (3)
N2—Mn1—O3—S1	−164.19 (11)	C24—N3—C13—C14	1.0 (3)
O1—Mn1—N1—C1	−76.97 (18)	Mn1—N3—C13—C14	−176.08 (17)
O3—Mn1—N1—C1	10.13 (18)	N3—C13—C14—C15	−2.5 (4)
N4—Mn1—N1—C1	92.10 (18)	C13—C14—C15—C16	1.3 (4)
N3—Mn1—N1—C1	169.89 (17)	C14—C15—C16—C24	1.0 (4)
N2—Mn1—N1—C1	−176.99 (19)	C14—C15—C16—C17	−179.7 (3)
O1—Mn1—N1—C12	97.39 (14)	C15—C16—C17—C18	−177.3 (3)
O3—Mn1—N1—C12	−175.52 (14)	C24—C16—C17—C18	2.0 (4)
N4—Mn1—N1—C12	−93.55 (14)	C16—C17—C18—C19	−0.3 (5)
N3—Mn1—N1—C12	−15.7 (2)	C17—C18—C19—C23	−0.7 (4)
N2—Mn1—N1—C12	−2.64 (13)	C17—C18—C19—C20	179.8 (3)
O1—Mn1—N2—C10	90.70 (19)	C23—C19—C20—C21	−0.6 (4)
O3—Mn1—N2—C10	−160.28 (17)	C18—C19—C20—C21	179.0 (3)
N4—Mn1—N2—C10	−76.07 (19)	C19—C20—C21—C22	0.7 (5)
N3—Mn1—N2—C10	−2.88 (19)	C23—N4—C22—C21	−1.7 (4)
N1—Mn1—N2—C10	−177.7 (2)	Mn1—N4—C22—C21	−178.3 (2)
O1—Mn1—N2—C11	−91.80 (15)	C20—C21—C22—N4	0.5 (5)
O3—Mn1—N2—C11	17.2 (2)	C22—N4—C23—C19	1.7 (3)
N4—Mn1—N2—C11	101.42 (14)	Mn1—N4—C23—C19	178.69 (18)
N3—Mn1—N2—C11	174.61 (15)	C22—N4—C23—C24	−178.3 (2)
N1—Mn1—N2—C11	−0.17 (13)	Mn1—N4—C23—C24	−1.4 (2)
O1—Mn1—N3—C13	−20.27 (18)	C20—C19—C23—N4	−0.6 (4)
O3—Mn1—N3—C13	−109.82 (17)	C18—C19—C23—N4	179.8 (2)
N4—Mn1—N3—C13	176.81 (19)	C20—C19—C23—C24	179.4 (2)
N1—Mn1—N3—C13	93.0 (2)	C18—C19—C23—C24	−0.1 (4)
N2—Mn1—N3—C13	80.47 (18)	C13—N3—C24—C16	1.5 (3)
O1—Mn1—N3—C24	162.55 (14)	Mn1—N3—C24—C16	178.94 (17)
O3—Mn1—N3—C24	73.01 (15)	C13—N3—C24—C23	−177.63 (19)
N4—Mn1—N3—C24	−0.37 (14)	Mn1—N3—C24—C23	−0.2 (2)
N1—Mn1—N3—C24	−84.2 (2)	C15—C16—C24—N3	−2.5 (3)
N2—Mn1—N3—C24	−96.71 (14)	C17—C16—C24—N3	178.1 (2)
O1—Mn1—N4—C22	120.9 (2)	C15—C16—C24—C23	176.6 (2)
O3—Mn1—N4—C22	56.7 (2)	C17—C16—C24—C23	−2.7 (3)
N3—Mn1—N4—C22	177.6 (2)	N4—C23—C24—N3	1.1 (3)
N1—Mn1—N4—C22	−26.5 (2)	C19—C23—C24—N3	−179.0 (2)
N2—Mn1—N4—C22	−98.9 (2)	N4—C23—C24—C16	−178.1 (2)
O1—Mn1—N4—C23	−55.7 (3)	C19—C23—C24—C16	1.8 (3)
O3—Mn1—N4—C23	−119.97 (16)	Mn1—O1—C25—O2	144.8 (3)
N3—Mn1—N4—C23	0.93 (15)	Mn1—O1—C25—C26	−39.7 (4)
N1—Mn1—N4—C23	156.92 (15)	O1—C25—C26—C31	−134.3 (2)
N2—Mn1—N4—C23	84.47 (15)	O2—C25—C26—C31	41.6 (3)
C12—N1—C1—C2	−0.2 (3)	O1—C25—C26—C27	46.9 (3)
Mn1—N1—C1—C2	174.10 (18)	O2—C25—C26—C27	−137.2 (2)
N1—C1—C2—C3	−0.5 (4)	C31—C26—C27—C28	−4.6 (3)
C1—C2—C3—C4	1.9 (4)	C25—C26—C27—C28	174.23 (19)
C2—C3—C4—C12	−2.5 (3)	C31—C26—C27—S1	176.47 (17)
C2—C3—C4—C5	176.8 (2)	C25—C26—C27—S1	−4.7 (3)
C3—C4—C5—C6	−178.0 (2)	O4—S1—C27—C28	−129.68 (16)
C12—C4—C5—C6	1.2 (3)	O5—S1—C27—C28	−5.45 (18)
C4—C5—C6—C7	−2.5 (4)	O3—S1—C27—C28	111.71 (16)
C5—C6—C7—C8	−178.0 (2)	O4—S1—C27—C26	49.3 (2)
C5—C6—C7—C11	0.3 (4)	O5—S1—C27—C26	173.55 (17)
C11—C7—C8—C9	−2.4 (3)	O3—S1—C27—C26	−69.29 (18)
C6—C7—C8—C9	176.0 (2)	C26—C27—C28—C29	3.1 (3)
C7—C8—C9—C10	0.7 (4)	S1—C27—C28—C29	−177.84 (16)
C11—N2—C10—C9	−1.7 (3)	C27—C28—C29—C30	1.7 (3)
Mn1—N2—C10—C9	175.73 (18)	C27—C28—C29—C32	−177.2 (2)
C8—C9—C10—N2	1.5 (4)	C28—C29—C30—C31	−5.0 (3)
C10—N2—C11—C7	−0.1 (3)	C32—C29—C30—C31	174.0 (2)
Mn1—N2—C11—C7	−177.91 (15)	C29—C30—C31—C26	3.5 (4)
C10—N2—C11—C12	−179.41 (19)	C27—C26—C31—C30	1.3 (3)
Mn1—N2—C11—C12	2.8 (2)	C25—C26—C31—C30	−177.6 (2)
C8—C7—C11—N2	2.1 (3)	C28—C29—C32—O6	−163.7 (2)
C6—C7—C11—N2	−176.2 (2)	C30—C29—C32—O6	17.3 (4)
C8—C7—C11—C12	−178.61 (19)	C28—C29—C32—O7	14.8 (3)
C6—C7—C11—C12	3.0 (3)	C30—C29—C32—O7	−164.1 (2)

### Hydrogen-bond geometry (Å, °)

**Table:**

*D*—H···*A*	*D*—H	H···*A*	*D*···*A*	*D*—H···*A*
O7—H7···O2^i^	0.82	1.72	2.517 (3)	165
C8—H8···O6^ii^	0.93	2.42	3.209 (4)	142
C18—H18···O5^iii^	0.93	2.46	3.306 (3)	151

Symmetry codes: (i) *x*−1, *y*, *z*; (ii) *x*+1, *y*+1, *z*; (iii) −*x*+1, −*y*+2, −*z*+1.
